# Effects of heat waves and cold spells on blood parameters: a cohort study of blood donors in Tianjin, China

**DOI:** 10.1265/ehpm.24-00023

**Published:** 2024-04-25

**Authors:** Yutong Gao, Yifan Liu, Jiayu He, Yin Zhang, Ting Wang, Lin Wu, Naixiu Sun, Tiange Fang, Hongjun Mao, Nai-jun Tang, Xi Chen

**Affiliations:** 1 Tianjin Key Laboratory of Urban Transport Emission Research, College of Environmental Sciences and Engineering, Nankai University, Tianjin 300071, China; 2 Department of Occupational and Environmental Health, School of Public Health, Tianjin Medical University, Tianjin, 300070, China; 3 Tianjin Key Laboratory of Environment, Nutrition, and Public Health, Tianjin Medical University, Tianjin, 300070, China; 4 Tianjin Blood Center, 424 Huanghe Road, Tianjin 300110, China

**Keywords:** Heat wave, Cold spell, Blood donors, Blood parameters, Generalized additive mixed model

## Abstract

**Background:**

With the increasing occurrence of extreme temperature events due to climate change, the attention has been predominantly focused on the effects of heat waves and cold spells on morbidity and mortality. However, the influence of these temperature extremes on blood parameters has been overlooked.

**Methods:**

We conducted a cohort study involving 2,752 adult blood donors in Tianjin, China, between January 18, 2013, and June 25, 2021. The generalized additive mixed model was used to investigate the effects and lagged effects of heat waves and cold spells on six blood parameters of blood donors, including alanine aminotransferase (ALT), white blood cell count (WBC), red blood cell count (RBC), hemoglobin (HB), hematocrit (HCT), and platelet count (PLT). Subgroup analyses were stratified by sex, age, and BMI.

**Results:**

Heat waves and cold spells are associated with changes in blood parameters, particularly HB and PLT. Heat waves increased HB and PLT, while cold spells increased HB and decreased PLT. The effect of heat waves is greater than that of cold spells. The largest effect of heat waves on HB and PLT occurred at lag1 with 2.6 g/L (95% *CI*: 1.76 to 3.45) and lag7 with 9.71 × 10^9/L (95% *CI*: 6.26 to 13.17), respectively, while the largest effect of cold spells on HB and PLT occurred at lag0 with 1.02 g/L (95% *CI*: 0.71 to 1.33) and lag2 with −3.85 × 10^9/L (95% *CI*: −5.00 to −2.70), respectively. In subgroup analysis, the effect of cold spells on ALT was greater in the 40–49 age group.

**Conclusion:**

We indicated that heat waves and cold spells can impact hemoglobin and platelet counts in the human body. These findings provide evidence linking heat waves or cold spells to diseases and may reduce health risks caused by extreme temperature events.

**Supplementary information:**

The online version contains supplementary material available at https://doi.org/10.1265/ehpm.24-00023.

## 1. Introduction

In the context of climate change, the global climate system is complex and unstable, leading to an increasing frequency and intensity of extreme weather events such as heat waves and cold waves. Many studies have demonstrated a correlation between ambient temperature and human health, such as a V-like relationship between mortality and temperature [1]. The drastic fluctuations in environmental temperature can cause various physiological and even pathological changes in the human body. Numerous studies have shown that extreme temperature events (heat waves and cold spells) increase the risk of morbidity and mortality, including cardiovascular diseases, respiratory diseases, digestive system diseases, neoplasms, and others [2–8]. Most of these studies have focused on morbidity and mortality as health outcomes, and the underlying biological process mediating the association between heat wave or cold spell exposure and the aforementioned diseases remains incompletely understood. However, changes in blood parameters are considered to be important driving factors [9–11].

Blood exhibits high fluidity, enabling constant exchange of substances and energy with tissues and organs. Therefore, blood parameters can serve as the “first window” reflecting overall health. Alanine aminotransferase (ALT) is a sensitive parameter of liver cell damage and is currently the most commonly used liver function test in clinical practice. ALT is mainly distributed in liver cells, and if the liver is damaged, transaminases in liver cells enter the blood, increasing ALT levels. White blood cell count (WBC) reflects the body’s immune status, red blood cells reflect the oxygen supply status, and platelets reflect the clotting status. Changes in these blood parameters can promptly indicate physiological or pathological changes occurring in the body, potentially triggering more severe diseases, such as cardiovascular diseases [12–14]. When exposed to extremely hot or cold environments, the human body’s thermoregulatory system helps maintain the balance of body temperature, resulting in changes in microcirculation, metabolic reactions, and collateral circulation, which can be directly manifested as changes in blood parameters. Therefore, analyzing the direct effects of extreme temperature weather on blood parameters is crucial for proactively preventing health risks associated with such climatic conditions. However, currently, there is a lack of relevant research, with only a few studies exploring the influence of environmental temperature on blood parameters. Temperature can affect blood biomarkers of coagulation and inflammation [15]. The relationship between environmental temperature and white blood cell (WBC) count shows a V-shaped curve, and when the temperature difference between adjacent days is large, WBC levels also increase [16]. Acute heat exposure can cause an increase in hematocrit, while chronic heat exposure can cause a decrease [17, 18]. Cold exposure can cause an increase in red blood cell count, hematocrit, and platelet count [19, 20]. The higher platelet count induced by the cold may be a potential cause of high winter cardiovascular disease mortality [10]. In addition, studies exploring the seasonal fluctuation of blood parameters showed that the changes in hemoglobin were related to temperature fluctuations [21], and ALT levels increased significantly in winter [22]. However, further research is needed to quantify the level of changes in blood parameters after exposure to extreme temperature weather, such as heat waves and cold spells.

Therefore, the purpose of this study was to investigate the effects and lagged effects of heat waves and cold spells on six blood parameters of the healthy population in Tianjin, China, through a long-term cohort study conducted between 2013 and 2021. Stratified analysis was also conducted to identify susceptible subgroups. Exploring the effects of extreme temperature weather on human blood parameters can timely reflect the physiological and pathological changes that occur in the body after exposure to heat waves and cold spells. The results will help develop strategies to cope with the health risks caused by extreme temperature weather in the future.

## 2. Methods

### 2.1. Study area and population

Tianjin (117.10°E, 39.10°N) is located on the west coast of the Pacific Ocean and in the northeast of the North China Plain. It has a warm temperate humid monsoon climate with four distinct seasons. During the time of the research, the temperature in this area ranges from −18.9 °C to 39.3 °C.

**Fig. 1:**
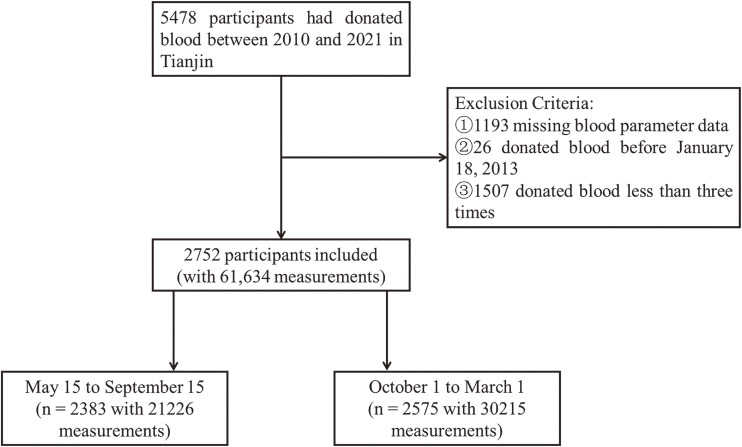
The flowchart for inclusion and exclusion of study participants.

This study was based on a cohort of blood donors that began on January 1, 2010, and followed up until June 25, 2021. A total of 5,478 adult participants who repeatedly donated blood components at the Tianjin Blood Center and resided in Tianjin were included. These blood donors must satisfy specific requirements: be aged between 18 and 60 years old, with a weight exceeding 50 kilograms for males and 45 kilograms for females, systolic blood pressure between 90 mmHg and 140 mmHg, diastolic blood pressure between 60 mmHg and 90 mmHg, a pulse pressure difference of at least 30 mmHg/4.0 kPa, a pulse rate ranging from 60 to 100 beats per minute, and a normal body temperature. This cohort represents a special population of regular component blood donors typically associated with poor living conditions and low socioeconomic status, mainly belonging to the lower-to-middle socioeconomic class. The follow-up intervals and total number of visits varied among participants. Between January 1, 2010, and June 25, 2021, a total of 5,478 participants aged 18 years or older were recruited, with information provided by the Tianjin Blood Center. Participants underwent a health consultation, a general physical examination, and blood testing at each blood donation. The health consultation excluded individuals with respiratory, circulatory, urinary, hematological, endocrine, allergic, mental, malignant tumors, and infectious diseases, as well as those with a history or current use of drug dependence, alcohol dependence, or drug abuse. The data collected included personal basic information such as ID number, age, gender, height, weight, education level, and geographical location, as well as six blood parameter values: alanine transaminase (ALT), white blood cell count (WBC), red blood cell count (RBC), hemoglobin (HB), hematocrit (HCT), and platelet count (PLT). The date of blood collection was also recorded. We identified blood donation measurement data based on unique resident ID numbers for long-term follow-up. There were missing covariate data before January 18, 2013, so we excluded participants with (1) missing blood parameter data, (2) blood donations before January 18th, 2013, and (3) less than three blood donations. Finally, a total of 2,752 individuals (with 61,634 measurements) were included in this study, of whom 2,383 (with 21,226 measurements) were admitted between May 15 and September 15, and 2,575 (with 30,215 measurements) were admitted between October and March (Fig. 1). The research project was approved by the Tianjin Medical University Research Ethics Committee, and we conducted all survey methods in accordance with the Helsinki Declaration Principles. Written informed consent of each subject was obtained prior to the study.

### 2.2. Blood parameter measurement

Participants were required to provide a blood sample via skin puncture prior to blood donation for blood testing, which included ALT, WBC, RBC, HB, HCT, and PLT. The blood samples collected from participants were analyzed using an automatic biochemical analyzer (Mindray BS-430, Mindray, Shenzhen, China) and a commercial analysis kit (Mindray 140122012, Mindray, Shenzhen, China) to measure ALT (U/L) using a continuous monitoring method; a fully automatic blood cell analyzer (Mindray BC-5180CRP, Mindray, Shenzhen, China) was used to obtain measurements of five indicators, including WBC (10^9/L), RBC (10^12/L), HB (g/L), HCT, and PLT (10^9/L), using a semiconductor laser, flow cytometry technology, and chemical staining at a constant temperature.

### 2.3. Environmental confounding factors

#### 2.3.1 Meteorological factors

The meteorological data during the study period were sourced from the Tianjin Meteorological Station, identified by WMO/ID = 54527, with coordinates at Latitude: 39.1 and Longitude: 117.167. The data were retrieved from http://rp5.ru/archive.php. The meteorological variables included daily minimum and maximum temperatures, hourly temperatures, relative humidity, atmospheric pressure, etc.

#### 2.3.2 Air pollution

Daily mass concentration data of particulate matter with an aerodynamic diameter of less than 2.5 µm (PM_2.5_) during the study period were acquired from the Tianjin Ecological Environment Monitoring Center. Initially, 24-hour average concentrations of PM_2.5_ were collected from the 21 air quality monitoring stations in Tianjin. Subsequently, the Baidu Maps Application Programming Interface (http://lbsyun.baidu.com/) was utilized to obtain (1) the geocoded longitude and latitude in WGS84 coordinates of individual residential addresses and (2) the absolute distance between the participant’s residential address and the nearby monitoring station. Secondly, we employed the Inverse Distance Weighting (IDW) model, in conjunction with PM_2.5_ data from air quality monitoring stations and residential addresses encoded in longitude and latitude, to calculate the daily average PM_2.5_ concentration exposed to each participant.

### 2.4. Definition of heat waves and cold spells

For heat waves and cold spells, there is no globally standardized definitions for them, and different regions employ varying criteria for definitions. Currently, the most widely adopted definition method encompass both threshold temperature and duration [23–26]. Considering that the heat wave only occurs in the warm months of the year and referring to the time when the Tianjin Meteorological Bureau issued the heat alert, we restricted the heat wave study period to warm days (May 15 to September 15). The daily average temperature was calculated based on the 24-hour mean value. Then, based on 3 temperature thresholds (95th, 97th, and 99th percentiles of the daily average temperature during the heat wave study period) and 3 durations (at least 2, 3, or 5 days), we defined 8 heat waves (The heat wave ≥ 99th and lasting for 5d did not occur). Similarly, we considered the cold months (October to March) as the cold spell study period. Then we defined cold spells according to 3 temperature thresholds (2nd, 5th, and 10th percentiles of the daily average temperature during the cold spell study period) and 3 durations (at least 2, 3, or 5 days), respectively, including 9 cold spell definitions.

### 2.5. Statistical analysis

Normal distribution tests were performed on all continuous variables. Continuous variables with a normal distribution were expressed as mean (standard deviation, SD); otherwise, they were expressed as median (P25, P75). While categorical variables were expressed in the figure (proportion).

In this study, longitudinal data on six blood parameters (ALT, WBC, RBC, HB, HCT, and PLT) were collected from blood donors. The initial blood donation time was set as 0, and time was defined as the interval between blood donations. Since the blood donation frequency and time intervals were different for each donor and the repeated measurements were autocorrelated, we used the generalized additive mixed model (GAMM) to analyze the relationship between extreme temperature weather exposure and the longitudinal blood parameters of component blood donors. Potential adjustments were made for sex (male vs. female), age (continuous variable), education level (junior high school or below vs. high school or vocational school vs. college or higher education), body mass index (BMI, normal weight vs. underweight vs. overweight vs. obesity), atmospheric pressure (continuous variable), relative humidity (continuous variable), and PM_2.5_ concentration (continuous variable) in all analyses. The blood donor ID was introduced into the model as a random variable. First, we fitted the relationship between extreme temperature weather exposure and longitudinal blood parameters separately for several different definitions of heat waves and cold spells and presented the results using regression coefficients (*β*) and 95% confidence intervals (*CI*s). Then, according to the Akaike information criterion, the definitions with the best fitting effect were used for further analyses. The impact of extreme temperature exposure on health usually exhibits a lag effect. In this study, we considered single-day lag (lag0, lag1, … lag7) and multi-day cumulative lag (lag0-1, lag0-2, … lag0-7) effects.

We further evaluated the modifying effects of individual characteristics at the lag day with the maximum effect, stratified by sex (male vs. female), age group (young: 18–29 years old vs. middle youth: 30–39 years old vs. middle-aged: 40–49 years old vs. elderly: 50–58 years old), and BMI (underweight: <18.5 vs. normal weight: 18.5–25 vs. overweight: 25–30 vs. obesity: ≥30 kg/m^2^). And the multiplicative interaction terms between exposure variables and stratification variables were constructed to test whether the effect values of different populations are consistent, that is, to test whether the interlayer differences are significant. If the effect values of different populations are heterogeneous, it indicates an interaction between stratification variables and exposure.

A two-tailed p value of less than 0.05 was considered statistically significant. All analyses were performed using R software (version 4.2.0).

## 3. Results

### 3.1. Descriptive analysis

Table 1 describes the characteristics of the participants included in our study and shows a summary of environmental exposure data. Of the 2752 participants eligible for analysis, 72.97% were male, with ages ranging from 18 to 58 years and a mean age of 30.5 years. The follow-up time ranged from 28 to 3078 days (mean: 1049 days). The average temperature during the heat wave study was 25.9 °C, and 2.8 °C during the cold wave study. Table 2 show the definitions and occurrence frequencies of heat waves and cold spells Tianjin from 2013 to 2021.

**Table 1:** Descriptive Statistics of Population Characteristics, Meteorological Factors, and PM_2.5_.

	**Overall** **(n = 2752)**	**Warm period^a^** **(n = 2383)**	**Cold period^a^** **(n = 2575)**
Sex, n (%)			
Male	2008 (72.97)	1769 (74.23)	1880 (73.01)
Female	744 (27.03)	614 (25.77)	695 (26.99)
Age (year)			
All, M (P25, P75)	32 (42–25)	32 (42–25)	32 (42–25)
18–29, n (%)	1458 (52.98)	1255 (52.66)	1364 (52.97)
30–39, n (%)	675 (24.53)	585 (24.55)	631 (24.50)
40–49, n (%)	469 (17.04)	405 (17.00)	436 (16.93)
≥50, n (%)	150 (5.45)	138 (5.79)	144 (5.59)
Education, n (%)			
College or University degree	1340 (48.69)	1128 (47.34)	1241 (48.19)
High school or Secondary school	618 (22.46)	553 (23.21)	581 (22.56)
Junior high or less	794 (28.85)	702 (29.46)	753 (29.24)
BMI (kg/m^2^), n (%)			
normal weight (18.5–25)	1550 (56.32)	1361 (57.11)	1447 (56.19)
underweight (<18.5)	92 (3.34)	75 (3.15)	73 (2.83)
overweight (25–30)	891 (32.38)	767 (32.19)	845 (32.82)
obesity (≥30)	219 (7.96)	180 (7.55)	210 (8.16)
Blood Parameter, mean ± SD			
ALT (U/L)	18.9 ± 9.7	18.8 ± 9.6	19.1 ± 9.7
WBC (10^9/L)	6.1 ± 1.7	6.2 ± 1.5	6.1 ± 1.8
RBC (10^12/L)	4.9 ± 0.4	4.9 ± 0.4	4.9 ± 0.4
HB (g/L)	145.3 ± 14.0	144.1 ± 13.7	146.4 ± 14.1
HCT (L/L)	0.4 ± 0.0	0.4 ± 0.0	0.4 ± 0.0
PLT (10^9/L)	268.7 ± 56.7	270.4 ± 56.6	267.2 ± 56.8
Ambient PM_2.5_ air pollution (µg/m^3^), M (P25, P75)	50.9 (82.5–31.3)	44.6 (61.3–30.5)	62.2 (107.1–32.6)
Meteorological Factor, mean ± SD			
Temperature (°C)	14.0 ± 11.1	25.9 ± 3.0	2.8 ± 5.6
Atmospheric pressure (mmHg)	761.8 ± 4.3	754.8 ± 3.5	768.7 ± 5.0
Relative humidity (%)	57.5 ± 17.1	63.0 ± 15.1	51.9 ± 19.0

^a^Warm period: May 15 to September 15; Cold period: October 1 to March 1.

Abbreviations: BMI, body mass index; ALT, alanine aminotransferase; WBC, white blood cell; RBC, red blood cell count; HB, hemoglobin; HCT, hematocrit; PLT, platelet count.

**Table 2:** Definitions and frequency of heat waves and cold spells in Tianjin.

**Heat ** **waves**	**Threshold percentile^a^** **(Threshold temperature (°C))**	**Duration** **(days)^b^**	**Number of days** **(%^c^)**
HW^e^01	95(30.71)	2	38(3.68)
HW02	95(30.71)	3	26(2.51)
HW03	95(30.71)	5	12(1.16)
HW04	97(31.30)	2	21(2.03)
HW05	97(31.30)	3	11(1.06)
HW06	97(31.30)	5	5(0.48)
HW07	99(32.06)	2	7(0.68)
HW08	99(32.06)	3	3(0.29)

**Cold ** **spells**	**Threshold percentile** **(Threshold temperature (°C))**	**Duration** **(days)**	**Number of days** **(%^d^)**

CS^e^01	2(−6.36)	2	26(1.70)
CS02	2(−6.36)	3	20(1.31)
CS03	2(−6.36)	5	7(0.46)
CS04	5(−4.18)	2	64(4.18)
CS05	5(−4.18)	3	52(3.40)
CS06	5(−4.18)	5	17(1.11)
CS07	10(−3.00)	2	128(8.36)
CS08	10(−3.00)	3	106(6.92)
CS09	10(−3.00)	5	65(4.25)

^a^Threshold temperature refers to the temperature value corresponding to the threshold percentage of the daily average temperature distribution during the study period

^b^Duration refers to the number of days that the daily average temperature continues to exceed the threshold temperature

^c^Proportion of heat waves during the heat wave study period (May 15 to September 15)

^d^Proportion of cold spells during the cold spell study period (October 1 to March 1)

^e^HW, heat wave; CS, cold spell

### 3.2. Associations of heat waves and cold spells with blood parameters

We used GAMM to analyze the relationship between the different definitions of heat waves and cold spells and the six blood parameters after adjusting for factors such as sex, age, education level, BMI, air pressure, relative humidity, and PM_2.5_ exposure concentration, as shown in Table 3. The effects of heat waves and cold spells under different definitions (HW01-HW08, CS01-CS09) on six blood parameters generally increased with the increase in definition intensity, and the confidence interval of the effect estimates widened with stricter definitions. Among the various definitions, HW05 (≥97th percentile for at least 3 days) and CS07 (≤10th percentile for at least 2 days) provided the best fit for the model. Therefore, we primarily present the health effects of heatwaves and cold spells based on HW05 and CS07, respectively.

**Table 3:** Associations of heat waves and cold spells by different definitions with six blood parameters.

**Heat waves**	**ALT**	**WBC**	**RBC**	**HB**	**HCT**	**PLT**

	***β* (95%*CI*^a^)**	***β* (95%*CI*)**	***β* (95%*CI*)**	***β* (95%*CI*)**	***β* (95%*CI*)**	***β* (95%*CI*)**
HW^a^01	0.35(−0.13,0.84)	0.00(−0.06,0.07)	0.00(−0.01,0.01)	0.09(−0.37,0.55)	−0.0001(−0.0014,0.0012)	5.42(3.59,7.25)***
HW02	0.21(−0.37,0.80)	−0.02(−0.10,0.07)	−0.02(−0.03,0.00)*	0.8(0.24,1.37)**	−0.0020(−0.0035,−0.0004)*	6.8(4.57,9.02)***
HW03	−0.72(−1.57,0.12)	−0.06(−0.18,0.06)	−0.01(−0.03,0.02)	0.85(0.03,1.66)*	0.0004(−0.0019,0.0026)	10.31(7.09,13.53)***
HW04	0.62(−0.02,1.26)	0.01(−0.08,0.10)	0.00(−0.02,0.02)	1.08(0.46,1.69)***	0.0008(−0.0009,0.0026)	5.64(3.20,8.07)***
HW05	0.51(−0.40,1.42)	−0.05(−0.18,0.08)	0.01(−0.01,0.04)	2.29(1.41,3.16)***	0.0019(−0.0006,0.0044)	10.52(7.06,13.98)***
HW06	−2.00(−4.65,0.65)	−0.33(−0.70,0.04)	−0.02(−0.09,0.06)	1.05(−1.50,3.60)	−0.0012(−0.0083,0.0060)	13.97(3.90,24.05)**
HW07	1.77(0.59,2.95)**	0.08(−0.08,0.24)	0.01(−0.02,0.04)	1.35(0.22,2.48)*	0.0016(−0.0016,0.0048)	2.00(−2.47,6.46)
HW08	1.46(−0.35,3.28)	0.19(−0.06,0.44)	0.02(−0.04,0.07)	2.27(0.52,4.02)*	−0.0012(−0.0062,0.0037)	0.56(−6.35,7.46)

**Cold spells**	***β* (95%*CI*)**	***β* (95%*CI*)**	***β* (95%*CI*)**	***β* (95%*CI*)**	***β* (95%*CI*)**	***β* (95%*CI*)**

CS^a^01	−0.20(−0.90,0.49)	0.05(−0.08,0.18)	0.03(0.01,0.05)**	1.27(0.59,1.95)***	0.0008(−0.0011,0.0027)	−0.31(−2.94,2.33)
CS02	−0.35(−1.12,0.43)	0.03(−0.11,0.18)	0.02(0.00,0.04)	1.64(0.88,2.41)***	−0.0003(−0.0024,0.0018)	−1.57(−4.52,1.38)
CS03	−0.48(−1.68,0.71)	0.02(−0.21,0.25)	−0.03(−0.06,0.01)	2.77(1.60,3.95)***	−0.0081(−0.0114,−0.0049)***	−4.71(−9.27,0.15)*
CS04	0.03(−0.39,0.46)	−0.01(−0.10,0.07)	0.01(0.00,0.03)*	1.38(0.96,1.79)***	0.0011(0.0000,0.0023)	−2.3(−3.92,−0.68)**
CS05	−0.19(−0.68,0.29)	0.03(−0.07,0.12)	0.03(0.02,0.05)***	1.02(0.54,1.50)***	0.0029(0.0015,0.0042)***	0.06(−1.79,1.92)
CS06	−0.21(−0.83,0.41)	0.02(−0.10,0.14)	0.01(−0.01,0.03)	0.73(0.12,1.34)*	−0.0002(−0.0018,0.0015)	−0.12(−2.48,2.24)
CS07	−0.15(−0.47,0.16)	−0.05(−0.11,0.01)	−0.01(−0.02,0.00)	1.02(0.71,1.33)***	−0.0012(−0.0021,−0.0004)**	−4.01(−5.20,−2.82)***
CS08	−0.08(−0.42,0.25)	−0.05(−0.11,0.02)	−0.01(−0.02,0.00)*	0.87(0.54,1.20)***	−0.0016(−0.0025,−0.0007)***	−4.46(−5.74,−3.18)***
CS09	−0.22(−0.62,0.18)	−0.05(−0.12,0.03)	−0.01(−0.02,0.00)	0.79(0.40,1.18)***	−0.0018(−0.0029,−0.0007)***	−3.99(−5.51,−2.47)***

^a^*CI*, confidence interval; HW, heat wave; CS, cold spell; ALT, alanine aminotransferase; WBC, white blood cell; RBC, red blood cell count; HB, hemoglobin; HCT, hematocrit; PLT, platelet count.

*P < 0.05, **P < 0.01, ***P < 0.001

**Fig. 2:**
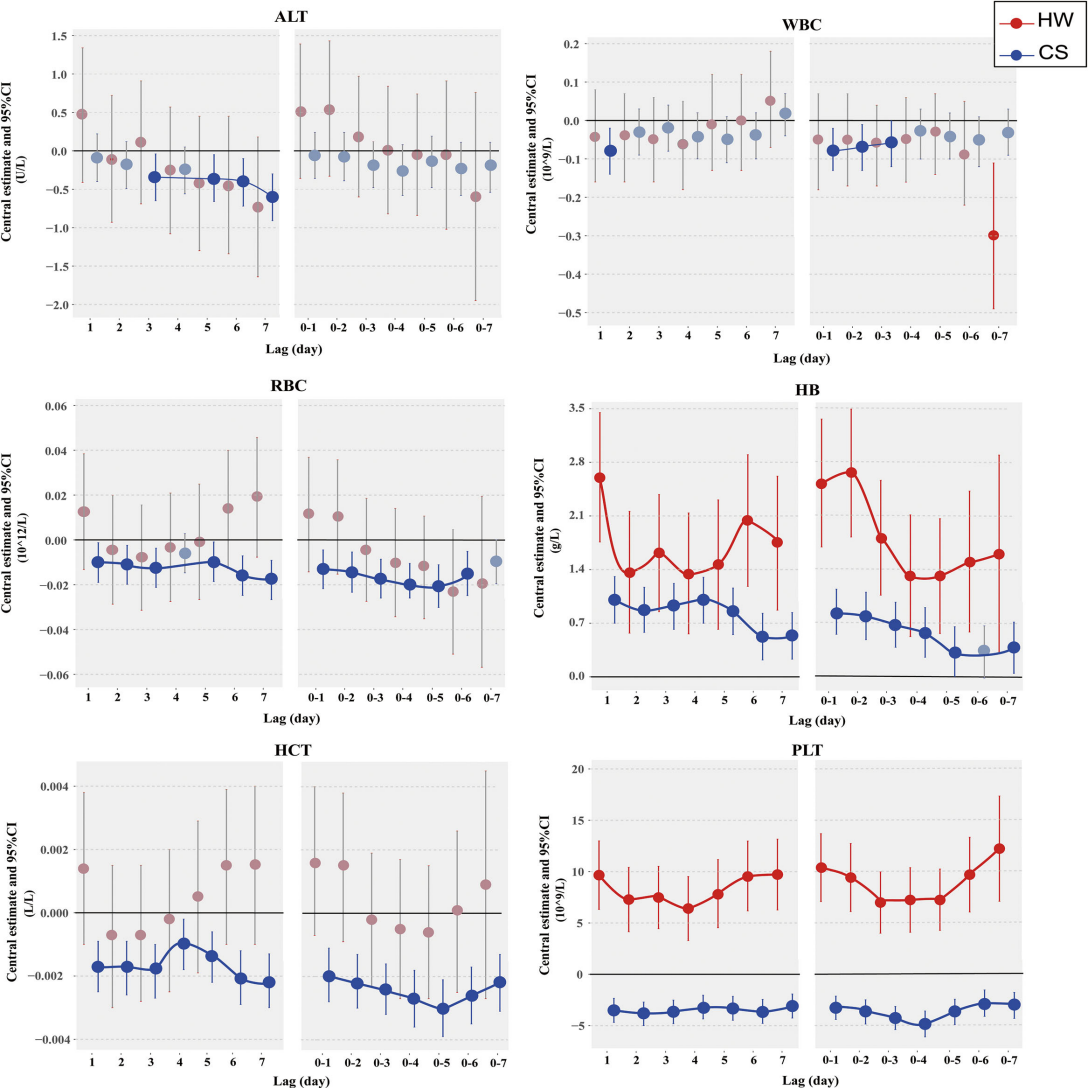
Single-day and cumulative lag effects of heat wave/cold spell on blood parameters. The best definitions HW05 and CS07 were used for heat waves and cold spells, respectively. Gray indicates no statistical significance. Abbreviations: CI, confidence interval; HW, heat wave; CS, cold spell; ALT, alanine aminotransferase; WBC, white blood cell; RBC, red blood cell count; HB, hemoglobin; HCT, hematocrit; PLT, platelet count.

Figure 2 shows the 1–7 single-day lag (lag1–lag7) and multi-day cumulative lag (lag0-1 ∼ lag0-7) effects of heat waves and cold spells on six blood parameters (with detailed numerical results presented in Tables S1–S2). The heat wave has a more significant impact on HB and PLT, whereas it does not have a significant effect on the other four parameters: ALT, WBC, RBC, and HCT. In lag studies, the cold wave shows statistically significant effects on all six parameters, indicating that, compared to heat waves, cold waves have a more widespread impact on human blood parameters. However, the effect of the cold wave on ALT, WBC, RBC, and HCT levels is relatively weaker. Overall, both heat waves and cold waves exposure affect blood parameters in the population, especially significantly impacting HB and PLT. Heatwaves increase both, while cold waves increase hemoglobin and decrease platelet count. Furthermore, both exhibit lagged effects. The largest effect of heat waves on HB occurred at lag1 day, causing an increase of 2.6 g/L (95% *CI*: 1.76 to 3.45), followed by a significant decrease, and the corresponding largest multi-day cumulative lag effect occurred at lag0-2. The effect of heat waves on PLT was more sustained, the largest effect occurred at lag7 day, increasing its value by 9.71 × 10^9/L (95% *CI*: 6.26 to 13.17), and the largest multi-day cumulative lag effect occurred at lag0-7. Cold spells increased the HB value, with the largest effect on it occurring on the day of the cold spell, increasing by 1.02 g/L (95% *CI*: 0.71 to 1.33), followed by a significant decrease. The largest multi-day cumulative lag effect occurred at lag0-1. Cold spells reduced PLT values, with the largest effect on PLT at lag2 day, which decreased by 3.85 × 10^9/L (95% *CI*: −5.00 to −2.70), with the largest multi-day cumulative lag effect occurring at lag0-4. Notably, for HB and PLT, the effects of heat waves were greater than those of cold spells. In addition, the lag effects of the cold spell on ALT were greater than the cold spell itself. On the third day following the cold spell, ALT decreased by −0.35 U/L (95% *CI*: −0.65 to −0.04), whereas on the day of the cold wave occurrence, the population’s ALT is not significantly affected (P = 0.3379).

### 3.3. Subgroups analysis

**Fig. 3:**
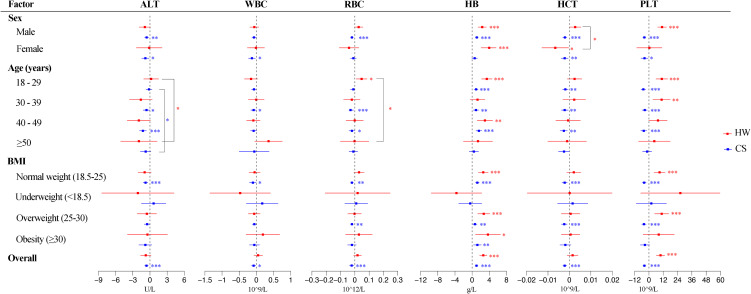
Impact of heat waves/cold spells on blood parameters in overall and subgroup. The best definitions HW05 and CS07 were used for heat waves and cold spells respectively. The stratified analyses of heat waves on ALT, WBC, RBC, HB, HCT, and PLT were carried out at lag7, lag4, lag7, lag1, lag7, and lag7, respectively; and cold spells on ALT, WBC, RBC, HB, HCT, and PLT were carried out at lag7, lag1, lag7, lag0, lag7, and lag2, respectively. *P < 0.05, **P < 0.01, ***P < 0.001, P value was calculated through the chi-square test. Abbreviations as in Fig. 2.

And we explored the modification effect of individual characteristics. Figure 3 displays the effects of heat waves and cold waves on the six blood parameters for all subgroups of the population on the lag-day with the maximum effects (detailed numerical information is provided in Tables S3–S4). Interaction tests showed that the effects of the cold spell on ALT was influenced by age (P = 0.0203), with the ALT of the 40–49 age group being more affected by the cold spell (*β*: −1.23; 95% *CI*: −1.83 to −0.64) than that of the 30–39 age group (*β*: −0.6; 95% *CI*: −1.18 to −0.02). There were no statistically significant differences in the effects of the cold spell on other parameters among different sex, age, or BMI groups (P > 0.05).

## 4. Discussion

Numerous studies indicate that climate change is expected to lead to an increasing frequency of extreme temperature events in the future. The resulting health risks from heat waves and cold spells are anticipated to escalate [27–29]. Prolonged exposure to high temperatures can easily disrupt human metabolism and immune function, resulting in symptoms such as fatigue, dizziness, nausea, and potentially serious consequences such as heat stroke and dehydration [30]. Cold spells may cause frostbite and chilblains, exacerbate the incidence of respiratory and cardiovascular diseases, and even lead to death [31]. Blood parameters are a window reflecting numerous physiological and pathological conditions, and observing changes in these parameters can provide timely warning and intervention for malignant diseases. However, to the best of our knowledge, there is currently no research on the effects of heat waves and cold spells on various blood parameters in humans. Additionally, other environmental factors besides temperature can also affect human health [32, 33], among which PM_2.5_ is considered the main air pollutant causing adverse health effects [34, 35]. Therefore, this study conducted quantitative analysis on the impact of extreme temperature heat waves and cold waves on various blood parameters in humans, filling the research gap in this field. And we also included atmospheric pressure, relative humidity, and PM_2.5_ concentration as confounding factors in the analysis.

There is no globally standardized definition for heat waves and cold spells, and due to different population characteristics in different regions, their definitions have regional heterogeneity. The most widely adopted definition method encompasses both threshold temperature and duration [23–26]. In addition, regarding the selection of temperature indicators, Anderson et al. [36] conducted a comparative analysis of the cold and heat effects when employing daily minimum, maximum, apparent, and mean temperatures as indicators. The findings revealed that the heat effect was most pronounced with mean daily temperature, aligning with results from a comprehensive European study [37]. Moreover, this research indicated that different temperature indicators are likely to yield similar outcomes. Consequently, this study opts for the mean daily temperature indicator and delineates multiple definitions for heat waves and cold spells through three threshold temperatures and three durations, attempting to find out the optimal definition of heat waves and cold waves associated with changes in blood parameters in Tianjin. We found that in most cases, the effect of heat waves (or cold spells) increases with the increase (or decrease) of threshold temperature and increased duration, which has been confirmed by previous studies [38–40]. In addition, we found that the definitions with the best model fit were HW05 (the 97th percentile and duration of at least 3 days) and CS07 (the 10th percentile threshold and duration of at least 2 days). Subsequently, this study explored the effects of heat waves and cold spells on six blood parameters in the blood donor population in Tianjin, China, using these definitions.

We found that heat waves and cold spells mainly affect HB and PLT. Specifically, exposure to heat waves increased both HB and PLT levels, while exposure to cold spells increased HB levels and decreased PLT levels. Consistent with our study, previous studies have shown that exposure to hot environments can increase PLT, RBC, and plasma viscosity [41], while Al-Otaibi et al. [42] observed significantly abnormal HB and PLT levels among individuals working in high-temperature environments compared to those in the control group. From a clinical perspective, these alterations in HB and PLT levels may reflect the body’s adaptation and regulation to extreme temperatures. This phenomenon may be due to the fact that high temperatures can cause sweating and dehydration, making the blood more viscous. Furthermore, the heightened metabolic rate in hot conditions increases oxygen demand, prompting the body to increase HB levels to enhance oxygen transport and meet tissue oxygen requirements. Simultaneously, changes in PLT levels signify an immune system response to external stimuli, suggesting that hot weather may activate the body’s inflammatory and immune systems [43]. These alterations may increase the cardiovascular system’s burden, potentially raising the risk of thrombosis and cardiovascular diseases. On the other hand, Checinska-Maciejewska et al. found that short-term exposure to severe cold stress, such as cold water swimming, increased HB levels and decreased PLT counts [44]. Exposure to low temperatures induces physiological stress, triggering hormone release like adrenaline. This, combined with the need to enhance oxygen-carrying capacity, result in an elevation of HB levels. The function of endothelial cells in blood vessels may also be impaired [45], leading to platelet aggregation or adhesion on the vessel wall, thereby reducing the number of free platelets in the blood and potentially causing coagulation disorders, which may increase the risk of bleeding.

Extreme temperature often exhibits lagged effects on population health. In previous studies focusing on mortality rates, it has been demonstrated that the impact of low temperatures on mortality persists for a longer duration, whereas the effects of heat are more immediate [46]. However, there is currently a lack of research analyzing the lag effects of extreme temperatures on human blood parameters. Our study findings reveal that within the 7 days following a heat wave or cold spell, both heat waves and cold spells significantly influence HB and PLT levels. Specifically, the peak effects of heat waves on HB and PLT occur at lag1 and lag7, respectively, while the peak effects of cold spells on HB and PLT occur at lag0 and lag2, respectively. According to the research results, extreme temperature events may have a relatively sustained impact on the human body, manifested specifically by a prolonged influence on HB and PLT levels. The appearance of peak effects during the lagged period may reflect the body’s physiological adaptation process to temperature changes, potentially involving internal regulatory mechanisms. Furthermore, although existing studies suggest that the impact of high-temperature heatwaves on population mortality or disease incidence is relatively short-term, our research indicates that the potential sustained effects of high-temperature heatwaves on human blood parameters should not be overlooked. In addition, the lag effects of cold spell exposure on ALT were greater than that of the cold spell itself, this means that even if individuals do not experience discomfort on the day of cold spell occurrence, they may still be affected by the lagged impact of cold spells, highlighting the need for continued vigilance and preventive measures. This finding is similar to the results of a study by Yan Wang et al. [26] in 2017, which found that the effects of cold spells on mortality increased with their duration and that the lag effects were greater than that of the cold spell itself.

We also found that, compared to cold spells, heat waves have a greater impact on blood parameters. The effects of heat waves and cold spells on different populations vary [6, 36, 47], and the spatial heterogeneity of this effect suggests that the relationship between extreme temperature weather and health in one community may not apply to another. Generally, people in warm regions have poorer cold resistance and are more susceptible to the effects of cold spells, while people in cold regions are more susceptible to the harm of heat waves. This study was conducted in Tianjin, which is located in northern China and has significant temperature variations and relatively cold temperatures. The study population comprised regular component blood donors. Given that regular component blood donors typically receive certain rewards, they are generally considered to have lower economic conditions and a diminished ability to cope with extreme temperature weather. Centralized heating is generally available in winter, but the coverage of heat protection measures for the summer is much lower. Therefore, the population in this study was more affected by heat waves than by cold spells.

We then stratified according to sex, age, and BMI to explore the differences in the effects of heat waves and cold spells on different populations. Due to the predominant inclusion of young and middle-aged individuals in this study, the sample size for individuals aged 50 and above was relatively limited. The research findings indicated that the influence of cold spells on ALT is age-dependent. Specifically, the impact is more pronounced in the 40–49 age group compared to the 30–39 age group, suggesting that older individuals are more susceptible to the effects of extreme temperature weather. Consistent with our study, some studies have also found that the elderly are more susceptible to the effects of heat waves and cold spells [36]. For example, a study conducted in China found that the effect of cold spells was stronger in the elderly aged ≥75 years than in those aged 0–64 years [48]. This may be due to the fact that the elderly usually have poor adaptation to extreme temperature environments, may suffer from certain chronic diseases, and have a lower risk perception for heat waves and cold spells [49, 50]. We did not observe significant differences according to the stratified analysis based on the sex and BMI of the population. Previous studies on the impact of heat waves and cold spells on gender stratification have not reached a unified conclusion [4, 51–54].

Our study suggests that public health authorities should proactively address the adverse impacts of extreme temperature weather. On the one hand, various targeted public awareness campaigns and educational initiatives should be implemented. These efforts should provide scientifically sound information on appropriate responses and self-rescue measures during extreme weather conditions, aiming to enhance the self-protective awareness of the general public, particularly among the elderly. On the other hand, in the event of a sustained trend of prolonged heatwaves and cold spells, corresponding early warning measures and comprehensive defense strategies should be implemented. This is crucial to mitigating the additional risks posed by prolonged extreme temperatures.

To our knowledge, this is the first report on the effects of heat waves and cold spells on various blood parameters in humans. Nevertheless, some limitations exist in this present study. Firstly, the target population of this study is the component blood donors, and due to the age limit for blood donation (18–60 years old) and the fact that most donors are in their youth, there is a lack of data on minors and individuals over the age of 60. Secondly, there is a lack of information on personal indoor exposure (such as the installation environment of air conditioning and heating) and lifestyle habits (smoking, diet, etc.). When extreme temperature weather occurs, people tend to stay indoors, and changes in diet would also affect changes in blood parameters, especially salt intake. In addition, daily physical activity can also affect human blood parameters, which may lead to an inaccurate assessment of the true impact of extreme temperature weather. Lastly, the meteorological data used in this study were collected from a limited number of surface meteorological stations. In the future, if conditions permit, we can consider acquiring more extensive meteorological data collected by satellites.

## 5. Conclusions

This long-term cohort study conducted between 2013 and 2021 on a healthy population revealed that heat waves and cold spells are associated with changes in blood parameters, particularly hemoglobin and platelet count. Heat waves increase both hemoglobin and platelet count, while cold spells increase hemoglobin but decrease platelet count. Moreover, the impact of heat waves was greater than that of cold spells. Older age groups appeared to be more susceptible to the effects of cold spells. In conclusion, in the future development of public health, attention should be paid to the occurrence of long-lasting and intense heatwaves and cold spells, and changes in various blood parameters in the human body should be monitored continuously after extreme temperature weather occurs. In the health risk research on climate change, attention should be paid to the protection of the elderly.

## Abbreviations

ALT: alanine aminotransferase
WBC: white blood cell count
RBC: red blood cell count
HB: hemoglobin
HCT: hematocrit
PLT: platelet count
CI: Confidence interval
WMO: World Meteorological Organization
PM_2.5_: particulate matter with an aerodynamic diameter of less than 2.5 µm
IDW: Inverse Distance Weighting
GAMM: generalized additive mixed model
BMI: body mass index
HW: heat wave
CS: cold spell

## Supplementary information

**Additional file 1:** **Table S1** Single-day lag effect of the heat wave, cold spell on six blood parameters in 1∼7 days. **Table S2** Multi-day cumulative lag effect of the heat wave, and cold spell on six blood parameters in 1∼7 days. **Table S3** Stratified analysis of the effects of heat waves on six blood parameters at the lag day with the largest effect. **Table S4** Stratified analysis of the effects of cold spells on six blood parameters at the lag day with the largest effect.

## Declarations

### Availability of data and material

Available upon reasonable request.
