# Supplementary material for: Effects of heat waves and cold spells on blood parameters: a cohort study of blood donors in Tianjin, China
Source: Environ Health Prev Med. 2024 Apr 25;29:25. doi: 10.1265/ehpm.24-00023 (PMC11058483; doi:10.1265/ehpm.24-00023)
Supplement: Supplementary file 1 — Additional file 1: Table S1 Single-day lag effect of the heat wave, cold spell on six blood parameters in 1∼7 days. Table S2 Multi-day cumulative lag effect of the heat wave, and cold spell on six blood parameters in 1∼7 days. Table S3 Stratified analysis of the effects of heat waves on six blood parameters at the lag day with the largest effect. Table S4 Stratified analysis of the effects of cold spells on six blood parameters at the lag day with the largest effect. [file ehpm-29-025-s001.docx]

**Supplemental Materials**

**Supplemental Tables**

**Table S1**

Single-day lag effect of the heat wave, cold spell on six blood parameters in 1~7 days.

**Table S2**

Multi-day cumulative lag effect of the heat wave, and cold spell on six blood parameters in 1~7 days.

**Table S3**

Stratified analysis of the effects of heat waves on six blood parameters at the lag day with the largest effect.

**Table S4**

Stratified analysis of the effects of cold spells on six blood parameters at the lag day with the largest effect.

**Table S1**

Single-day lag effect of the heat wave, cold spell on six blood parameters in 1~7 days.

|  | ALT | | WBC | | RBC | | HB | | HCT | | PLT | |
| --- | --- | --- | --- | --- | --- | --- | --- | --- | --- | --- | --- | --- |
| HW05 | β(95%CI) | P value | β(95%CI) | P value | β(95%CI) | P value | β(95%CI) | P value | β(95%CI) | P value | β(95%CI) | P value |
| lag1 | 0.47(-0.41,1.34) | 0.2965 | -0.04(-0.16,0.08) | 0.5358 | 0.0126(-0.0132,0.0384) | 0.3393 | 2.60(1.76,3.45)*** | <0.001 | 0.0014(-0.0010,0.0038) | 0.251 | 9.66(6.32,13.00)*** | <0.001 |
| lag2 | -0.11(-0.93,0.72) | 0.79998 | -0.04(-0.16,0.07) | 0.4787 | -0.0044(-0.0287,0.0199) | 0.7220 | 1.36(0.57,2.16)*** | <0.001 | -0.0007(-0.0030,0.0015) | 0.52 | 7.28(4.14,10.42)*** | <0.001 |
| lag3 | 0.11(-0.69,0.91) | 0.7865 | -0.05(-0.16,0.06) | 0.3767 | -0.0079(-0.0314,0.0156) | 0.5103 | 1.62(0.85,2.38)*** | <0.001 | -0.0007(-0.0028,0.0015) | 0.5548 | 7.48(4.44,10.52)*** | <0.001 |
| lag4 | -0.26(-1.08,0.57) | 0.5387 | -0.06(-0.18,0.05) | 0.2817 | -0.0033(-0.0275,0.0209) | 0.7870 | 1.35(0.56,2.14)*** | <0.001 | -0.0002(-0.0025,0.0020) | 0.8312 | 6.41(3.28,9.53)*** | <0.001 |
| lag5 | -0.42(-1.30,0.45) | 0.3445 | -0.01(-0.13,0.12) | 0.9252 | -0.0009(-0.0266,0.0249) | 0.9462 | 1.47(0.62,2.31)*** | <0.001 | 0.0005(-0.0019,0.0029) | 0.678 | 7.86(4.53,11.18)*** | <0.001 |
| lag6 | -0.45(-1.34,0.45) | 0.331 | 0.00(-0.13,0.12) | 0.9929 | 0.0135(-0.0128,0.0399) | 0.3146 | 2.04(1.18,2.90)*** | <0.001 | 0.0015(-0.0010,0.0039) | 0.2337 | 9.57(6.17,12.98)*** | <0.001 |
| lag7 | -0.73(-1.64,0.18) | 0.1161 | 0.05(-0.07,0.18) | 0.4064 | 0.019(-0.0077,0.0457) | 0.1627 | 1.75(0.87,2.62)*** | <0.001 | 0.0015(-0.0010,0.0040) | 0.2335 | 9.71(6.26,13.17)*** | <0.001 |
| CS07 | β(95%CI) | P value | β(95%CI) | P value | β(95%CI) | P value | β(95%CI) | P value | β(95%CI) | P value | β(95%CI) | P value |
| lag1 | -0.09(-0.40,0.22) | 0.5884 | -0.08(-0.14,-0.02)* | 0.0113 | -0.0101(-0.019,-0.0012)* | 0.0266 | 1.01(0.70,1.31)*** | <0.001 | -0.0017(-0.0025,-0.0009)*** | <0.001 | -3.51(-4.69,-2.33)*** | <0.001 |
| lag2 | -0.18(-0.49,0.12) | 0.238 | -0.03(-0.09,0.03) | 0.3104 | -0.0111(-0.0198,-0.0024)* | 0.0129 | 0.88(0.58,1.17)*** | <0.001 | -0.0017(-0.0026,-0.0009)*** | <0.001 | -3.85(-5.00,-2.70)*** | <0.001 |
| lag3 | -0.35(-0.65,-0.04)* | 0.0262 | -0.02(-0.08,0.04) | 0.4486 | -0.0124(-0.0212,-0.0037)** | 0.0054 | 0.92(0.62,1.22)*** | <0.001 | -0.0018(-0.0027,-0.0010)*** | <0.001 | -3.66(-4.82,-2.51)*** | <0.001 |
| lag4 | -0.25(-0.56,0.05) | 0.1067 | -0.04(-0.10,0.02) | 0.1887 | -0.006(-0.0147,0.0028) | 0.1840 | 1.00(0.70,1.30)*** | <0.001 | -0.001(-0.0018,-0.0002)* | 0.0167 | -3.19(-4.35,-2.03)*** | <0.001 |
| lag5 | -0.36(-0.66,-0.05)* | 0.0231 | -0.05(-0.11,0.01) | 0.1007 | -0.0098(-0.0186,-0.0009)* | 0.0305 | 0.85(0.55,1.16)*** | <0.001 | -0.0014(-0.0022,-0.0006)*** | <0.001 | -3.31(-4.48,-2.14)*** | <0.001 |
| lag6 | -0.41(-0.72,-0.10)** | 0.0093 | -0.04(-0.10,0.02) | 0.1942 | -0.0159(-0.0248,-0.0071)*** | <0.001 | 0.53(0.22,0.83)*** | <0.001 | -0.0021(-0.0029,-0.0012)*** | <0.001 | -3.61(-4.79,-2.44)*** | <0.001 |
| lag7 | -0.60(-0.91,-0.30)*** | <0.001 | 0.02(-0.04,0.07) | 0.6055 | -0.0179(-0.0267,-0.0091)*** | <0.001 | 0.54(0.23,0.84)*** | <0.001 | -0.0022(-0.0030,-0.0013)*** | <0.001 | -3.09(-4.26,-1.92)*** | <0.001 |

Abbreviations as in Table S2.

* P<0.05,** P<0.01,*** P<0.001

**Table S2**

Multi-day cumulative lag effect of the heat wave, and cold spell on six blood parameters in 1~7 days.

|  | ALT | | WBC | | RBC | | HB | | HCT | | PLT | |
| --- | --- | --- | --- | --- | --- | --- | --- | --- | --- | --- | --- | --- |
| HW05 | β(95%CI) | P value | β(95%CI) | P value | β(95%CI) | P value | β(95%CI) | P value | β(95%CI) | P value | β(95%CI) | P value |
| lag0-1 | 0.51(-0.36,1.39) | 0.2514 | -0.05(-0.18,0.07) | 0.3746 | 0.0114(-0.0142,0.0369) | 0.3825 | 2.53(1.70,3.37)*** | <0.001 | 0.0016(-0.0007,0.004) | 0.1720 | 10.30(6.99,13.61)*** | <0.001 |
| lag0-2 | 0.55(-0.33,1.43) | 0.2186 | -0.05(-0.17,0.07) | 0.3952 | 0.0103(-0.0152,0.0358) | 0.4276 | 2.67(1.83,3.50)*** | <0.001 | 0.0015(-0.0009,0.0038) | 0.2160 | 9.33(6.01,12.65)*** | <0.001 |
| lag0-3 | 0.18(-0.60,0.97) | 0.6498 | -0.06(-0.17,0.04) | 0.2485 | -0.0043(-0.0273,0.0186) | 0.7122 | 1.82(1.07,2.57)*** | <0.001 | -0.0002(-0.0023,0.0019) | 0.8732 | 6.90(3.91,9.89)*** | <0.001 |
| lag0-4 | 0.01(-0.82,0.84) | 0.9747 | -0.05(-0.16,0.06) | 0.3930 | -0.01(-0.0342,0.0141) | 0.4160 | 1.33(0.53,2.12)** | 0.0011 | -0.0005(-0.0027,0.0017) | 0.6709 | 7.15(4.00,10.30)*** | <0.001 |
| lag0-5 | -0.05(-0.84,0.74) | 0.9011 | -0.03(-0.14,0.07) | 0.5374 | -0.0122(-0.0351,0.0107) | 0.2978 | 1.32(0.57,2.07)*** | <0.001 | -0.0006(-0.0027,0.0015) | 0.5843 | 7.18(4.18,10.17)*** | <0.001 |
| lag0-6 | -0.05(-1.02,0.91) | 0.9135 | -0.09(-0.22,0.05) | 0.2060 | -0.0231(-0.0509,0.0047) | 0.1037 | 1.51(0.59,2.43)** | 0.0013 | 0.0001(-0.0025,0.0026) | 0.9554 | 9.61(5.97,13.26)*** | <0.001 |
| lag0-7 | -0.59(-1.95,0.76) | 0.3915 | -0.3(-0.49,-0.11)** | 0.0017 | -0.0196(-0.0586,0.0194) | 0.3256 | 1.60(0.31,2.90)* | 0.0155 | 0.0009(-0.0027,0.0045) | 0.6210 | 12.15(7.02,17.28)*** | <0.001 |
| CS07 | β(95%CI) | P value | β(95%CI) | P value | β(95%CI) | P value | β(95%CI) | P value | β(95%CI) | P value | β(95%CI) | P value |
| lag0-1 | -0.06(-0.36,0.24) | 0.7053 | -0.08(-0.13,-0.02)** | 0.0092 | -0.013(-0.0217,-0.0044)** | 0.0031 | 0.85(0.56,1.15)*** | <0.001 | -0.002(-0.0028,-0.0011)*** | <0.001 | -3.35(-4.49,-2.21)*** | <0.001 |
| lag0-2 | -0.08(-0.39,0.24) | 0.6384 | -0.07(-0.13,-0.01)* | 0.0254 | -0.0143(-0.0233,-0.0053)** | 0.0019 | 0.80(0.49,1.11)*** | <0.001 | -0.0022(-0.003,-0.0013)*** | <0.001 | -3.76(-4.94,-2.57)*** | <0.001 |
| lag0-3 | -0.18(-0.48,0.12) | 0.2424 | -0.06(-0.12,0.00)* | 0.0358 | -0.0172(-0.0259,-0.0085)*** | <0.001 | 0.69(0.39,0.98)*** | <0.001 | -0.0024(-0.0032,-0.0016)*** | <0.001 | -4.34(-5.48,-3.21)*** | <0.001 |
| lag0-4 | -0.25(-0.58,0.08) | 0.1421 | -0.03(-0.1,0.03) | 0.2923 | -0.0199(-0.0295,-0.0104)*** | <0.001 | 0.58(0.26,0.91)*** | <0.001 | -0.0027(-0.0036,-0.0018)*** | <0.001 | -4.93(-6.19,-3.66)*** | <0.001 |
| lag0-5 | -0.14(-0.48,0.19) | 0.3997 | -0.04(-0.1,0.02) | 0.2172 | -0.0206(-0.0301,-0.011)*** | <0.001 | 0.33(0.01,0.66)* | 0.046 | -0.003(-0.0039,-0.0021)*** | <0.001 | -3.76(-5.00,-2.51)*** | <0.001 |
| lag0-6 | -0.23(-0.58,0.11) | 0.1846 | -0.05(-0.12,0.01) | 0.1270 | -0.0149(-0.0248,-0.005)** | 0.0031 | 0.33(-0.01,0.67) | 0.0547 | -0.0026(-0.0035,-0.0017)*** | <0.001 | -2.90(-4.19,-1.61)*** | <0.001 |
| lag0-7 | -0.20(-0.54.0.14) | 0.2512 | -0.03(-0.09,0.03) | 0.3425 | -0.0097(-0.0195,0.0001) | 0.0526 | 0.38(0.05,0.72)* | 0.0238 | -0.0022(-0.0031,-0.0013)*** | <0.001 | -3.12(-4.39,-1.85)*** | <0.001 |

Abbreviations as in Table S2.

* P<0.05,** P<0.01,*** P<0.001

**Table S3**

Stratified analysis of the effects of heat waves on six blood parameters at the lag day with the largest effect.

|  | **ALT** | | **WBC** | | **RBC** | | **HB** | | **HCT** | | **PLT** | |
| --- | --- | --- | --- | --- | --- | --- | --- | --- | --- | --- | --- | --- |
| **Factor** | **β(95%CI)** | **P value** | **β(95%CI)** | **P value** | **β(95%CI)** | **P value** | **β(95%CI)** | **P value** | **β(95%CI)** | **P value** | **β(95%CI)** | **P value** |
| **Sex** |  | 0.5343^a^ |  | 0.8314^a^ |  | 0.1140^a^ |  | 0.0527^a^ |  | 0.0206^a^ * |  | 0.0522^a^ |
| Male | -0.89(-1.89,0.11) | 0.0805 | -0.06(-0.19,0.07) | 0.3616 | 0.027(-0.002,0.056) | 0.0699 | 2.35(1.41,3.28)*** | <0.001 | 0.0027(0.0000,0.0054) | 0.0506 | 10.98(7.36,14.61)*** | <0.001 |
| Female | -0.13(-2.37,2.11) | 0.9094 | -0.01(-0.26,0.24) | 0.9224 | -0.04(-0.108,0.027) | 0.2428 | 3.98(2.11,5.85)*** | <0.001 | -0.0066(-0.0128,-0.0004)* | 0.0382 | 0.33(-10.23,10.9) | 0.9507 |
| **Age** |  | 0.0149^a^ * |  | 0.2830^a^ |  | 0.0109^a^ * |  | 0.0702^a^ |  | 0.3132^a^ |  | 0.4004^a^ |
| 18-29 | 0.21(-1.08,1.49) | 0.7532 | -0.15(-0.35,0.04) | 0.1155 | 0.048(0.01,0.085)* | 0.0124 | 3.42(2.21,4.64)*** | <0.001 | 0.0024(-0.0009,0.0058) | 0.1585 | 10.79(5.94,15.63)*** | <0.001 |
| 30-39 | -1.6(-3.58,0.39) | 0.1148 | -0.01(-0.23,0.22) | 0.9651 | -0.021(-0.078,0.036) | 0.4750 | 1.26(-0.44,2.96) | 0.1474 | 0.0022(-0.003,0.0075) | 0.405 | 10.62(3.37,17.88)** | 0.0041 |
| 40-49 | -1.94(-3.97,0.08) | 0.0604 | -0.09(-0.28,0.11) | 0.3788 | -0.001(-0.061,0.059) | 0.9810 | 2.99(1.19,4.79)** | 0.0012 | -0.0006(-0.0063,0.0051) | 0.83504 | 7.57(-0.1,15.24) | 0.0531 |
| ≥50 | -1.94(-5.11,1.24) | 0.2321 | 0.36(-0.04,0.76) | 0.0820 | -0.002(-0.101,0.097) | 0.9739 | 1.37(-2.02,4.77) | 0.4277 | -0.0011(-0.0101,0.0079) | 0.819 | 4.5(-8.71,17.72) | 0.5042 |
| **BMI** |  | 0.6223^a^ |  | 0.6638^a^ |  | 0.3641^a^ |  | 0.7666^a^ |  | 0.5759^a^ |  | 0.8903^a^ |
| normal weight (18.5-25) | -0.93(-2.07,0.22) | 0.1140 | -0.05(-0.21,0.11) | 0.5319 | 0.03(-0.006,0.065) | 0.0996 | 2.59(1.45,3.74)*** | <0.001 | 0.0021(-0.0012,0.0053) | 0.214 | 8.92(4.36,13.49)*** | <0.001 |
| underweight (<18.5) | -2.11(-8.42,4.2) | 0.5128 | -0.47(-1.35,0.41) | 0.2951 | 0.02(-0.207,0.247) | 0.8644 | -3.65(-9.52,2.22) | 0.2230 | 0.0001(-0.0196,0.0199) | 0.9906 | 26.34(-6.88,59.55) | 0.1210 |
| overweight (25-30) | -0.54(-2.23,1.15) | 0.5308 | -0.06(-0.23,0.11) | 0.4706 | -0.002(-0.049,0.045) | 0.9370 | 2.71(1.3,4.12)*** | <0.001 | 0.0006(-0.0038,0.0049) | 0.802 | 10.68(4.85,16.52)*** | <0.001 |
| obesity (≥30) | -0.44(-3.93,3.06) | 0.8057 | 0.19(-0.30,0.68) | 0.4444 | 0.028(-0.065,0.122) | 0.5490 | 3.69(0.79,6.59)* | 0.0127 | 0.0027(-0.0056,0.0111) | 0.5241 | 8.2(-4.89,21.29) | 0.2199 |
| **Overall** | -0.73(-1.64,0.18) | 0.1161 | 0.05(-0.07,0.18) | 0.4064 | 0.019(-0.0077,0.0457) | 0.1627 | 2.60(1.76,3.45)*** | <0.001 | 0.0015(-0.0010,0.0040) | 0.2335 | 9.71(6.26,13.17)*** | <0.001 |

^a^ P value for interaction.

Abbreviations as in Table S2.

* P<0.05,** P<0.01,*** P<0.001

**Table S4**

Stratified analysis of the effects of cold spells on six blood parameters at the lag day with the largest effect.

|  | **ALT** | | **WBC** | | **RBC** | | **HB** | | **HCT** | | **PLT** | |
| --- | --- | --- | --- | --- | --- | --- | --- | --- | --- | --- | --- | --- |
| **Factor** | **β(95%CI)** | **P value** | **β(95%CI)** | **P value** | **β(95%CI)** | **P value** | **β(95%CI)** | **P value** | **β(95%CI)** | **P value** | **β(95%CI)** | **P value** |
| **Sex** |  | 0.5617^a^ |  | 0.5630^a^ |  | 0.7184^a^ |  | 0.0977^a^ |  | 0.6690^a^ |  | 0.8652^a^ |
| Male | -0.58(-0.93,-0.23)** | 0.0011 | -0.07(-0.13,0.002) | 0.0570 | -0.02(-0.03,-0.01)*** | <0.001 | 1.09(0.75,1.43)*** | <0.001 | -0.0021(-0.0031,-0.0012)*** | <0.001 | -3.87(-5.13,-2.61)*** | <0.001 |
| Female | -0.8(-1.42,-0.18)* | 0.0120 | -0.13(-0.23,-0.03)* | 0.0101 | -0.01(-0.03,0.01) | 0.2538 | 0.59(-0.06,1.23) | 0.0735 | -0.0022(-0.0038,-0.0006)** | 0.0087 | -3.73(-6.62,-0.83)* | 0.0116 |
| **Age** |  | 0.0203^a^ * |  | 0.9439^a^ |  | 0.1690^a^ |  | 0.3070^a^ |  | 0.4060^a^ |  | 0.3791^a^ |
| 18-29 | -0.17(-0.69,0.34) | 0.5126 | -0.07(-0.14,0.0016) | 0.0553 | -0.01(-0.03,0) | 0.1010 | 0.93(0.48,1.38)*** | <0.001 | -0.0019(-0.0032,-0.0006)** | 0.0050 | -4.72(-6.59,-2.85)*** | <0.001 |
| 30-39 | -0.6(-1.18,-0.02)* | 0.0433 | -0.08(-0.16,-0.0007)* | 0.0480 | -0.03(-0.04,-0.01)*** | <0.001 | 0.9(0.31,1.49)** | 0.0028 | -0.0022(-0.0037,-0.0006)** | 0.0068 | -3.51(-5.58,-1.45)*** | <0.001 |
| 40-49 | -1.23(-1.83,-0.64)*** | <0.001 | -0.08(-0.17,0.01) | 0.0749 | -0.02(-0.04,0)* | 0.0130 | 1.58(0.91,2.25)*** | <0.001 | -0.0026(-0.0043,-0.001)** | 0.0019 | -4.35(-6.81,-1.9)*** | <0.001 |
| ≥50 | -0.75(-1.67,0.16) | 0.1062 | -0.06(-0.5,0.37) | 0.7799 | -0.01(-0.04,0.02) | 0.3620 | 0.41(-0.67,1.49) | 0.4592 | -0.0025(-0.0051,0.0001) | 0.0585 | -1.52(-5.34,2.29) | 0.4332 |
| **BMI** |  | 0.4704^a^ |  | 0.5814^a^ |  | 0.9040^a^ |  | 0.0834^a^ |  | 0.9586^a^ |  | 0.7619^a^ |
| normal weight (18.5-25) | -0.77(-1.18,-0.37)*** | <0.001 | -0.1(-0.2,-0.0048)* | 0.0399 | -0.02(-0.03,-0.01)** | 0.0016 | 1.21(0.78,1.65)*** | <0.001 | -0.0022(-0.0034,-0.001)*** | <0.001 | -4(-5.65,-2.35)*** | <0.001 |
| underweight (<18.5) | 0.64(-1.5,2.78) | 0.5572 | 0.17(-0.29,0.63) | 0.4760 | 0.01(-0.07,0.09) | 0.8411 | -0.45(-3.13,2.23) | 0.7434 | 0.0015(-0.0055,0.0086) | 0.6718 | 2(-10.61,14.61) | 0.7560 |
| overweight (25-30) | -0.49(-1,0.02) | 0.0611 | -0.06(-0.12,0.01) | 0.0962 | -0.02(-0.03,-0.01)** | 0.0038 | 0.65(0.17,1.13)** | 0.0079 | -0.0024(-0.0037,-0.0011)*** | <0.001 | -4.21(-6.01,-2.41)*** | <0.001 |
| obesity (≥30) | -0.8(-1.92,0.32) | 0.1608 | -0.06(-0.2,0.09) | 0.4427 | -0.02(-0.05,0.01) | 0.2662 | 1.23(0.34,2.12)** | 0.0067 | -0.0019(-0.0044,0.0007) | 0.1477 | -3.39(-7.26,0.49) | 0.0869 |
| **Overall** | -0.60(-0.91,-0.30)*** | <0.001 | -0.08(-0.14,-0.02)* | 0.0113 | -0.0179(-0.0267,-0.0091)*** | <0.001 | 1.02(0.71,1.33)*** | <0.001 | -0.0022(-0.0030,-0.0013)*** | <0.001 | -3.85(-5.00,-2.70)*** | <0.001 |

^a^ P value for interaction.

Abbreviations as in Table S2.

* P<0.05,** P<0.01,*** P<0.001
